# Neuralgia

**Published:** 1851-07

**Authors:** 


*Neuralgia.*—Chloroform is now being used other than as an anæsthetic agent, and would seem highly efficacious as an ointment in neuralgic disturbances. Judging from what is said in French journals, the best, simplest, and most reliable preparation appears to be in the following formula:

| | |
|---|---|
| Chloroform, . . . . . . . . | 60 drops. |
| Hogs lard, . . . . . . . . | 1 oz. |

Mix in a mortar, and use several times per day, by rubbing on the affected part. Many forms of neuralgia resisting all the means used to subdue them, were said to yield kindly under the application of this. We intend giving it a fair trial, and will report; which we hope will be done by some of our readers also.

